# Thyroid Disease and Systemic Lupus Erythematosus

**DOI:** 10.3390/medicina59111911

**Published:** 2023-10-29

**Authors:** Lambros Athanassiou, Ifigenia Kostoglou-Athanassiou, Georgia Kaiafa, Pavlos Tsakiridis, Nikolaos Koukosias, Spyridon Mitsoulis, Christos Savopoulos, Panagiotis Athanassiou

**Affiliations:** 1 Department of Rheumatology, Asclepeion Hospital, Voula, 16673 Athens, Greece; lambros.ath@gmail.com; 2 Department of Endocrinology, Asclepeion Hospital, Voula, 16673 Athens, Greece; ikostoglouathanassiou@yahoo.gr; 3 First Propaedeutic Department of Internal Medicine, AHEPA University General Hospital, Aristotle University of Thessaloniki, 54636 Thessaloniki, Greece; gkaiafa@yahoo.gr (G.K.); chrisavopoulos@gmail.com (C.S.); 4 Department of Rheumatology, St. Paul’s Hospital, 55134 Thessaloniki, Greece; tsakiridis.p@gmail.com (P.T.); nikolaos_koukosias@yahoo.com (N.K.); spmitsoul@gmail.com (S.M.)

**Keywords:** systemic lupus erythematosus, autoimmune thyroid disease, hypothyroidism, hyperthyroidism, thyroid peroxidase antibodies, thyroglobulin antibodies

## Abstract

*Background and Objectives*: Thyroid disease has been associated with autoimmune disorders. As systemic lupus erythematosus (SLE) is a systemic autoimmune disease with diverse manifestations spanning across all organ systems, the relationship of SLE with thyroid disorders needs investigation. In particular, the relationship of SLE with autoimmune thyroid disease has attracted the interest of the research community. The aim was to evaluate the relationship of SLE with autoimmune thyroid disease. *Materials and Methods*: A cohort of 45 consecutive patients with a mean age of 47.97 years (range 21–79 years) and 45 age- and sex-matched controls were prospectively studied over a period of 12 months for the presence of thyroid disease and the prevalence of antithyroid antibodies. *Results*: Four patients (8.9%) were found to suffer from primary hypothyroidism, five (11.11%) from subclinical hypothyroidism and one (2.22%) from hyperthyroidism, whereas one (2.22%) of the controls had primary hypothyroidism and one (2.22%) had hyperthyroidism. Five patients (11.11%) had a thyroid hormone profile that was compatible with the presence of euthyroid sick syndrome. Thyroid peroxidase (TPOab) and thyroglobulin (Tgab) antibodies were detected in 20/45 and 15/45 of the SLE population and in 7/45 and 5/45 of the controls, respectively (*p* < 0.05, chi-square test). *Conclusions*: In conclusion, the incidence of clinical thyroid disease is greater amongst SLE patients than in a control population, and in a significant number of these patients, antithyroid antibodies are detectable. Thus, a subset of lupus patients appears to be predisposed to the development of thyroid disease, and this should be considered when evaluating patients with SLE.

## 1. Introduction

Systemic lupus erythematosus (SLE) is a systemic autoimmune disease that affects all organ systems [1]. It affects the skin, the joints, the vascular system and the heart, and may have hematological manifestations [2,3]. The disease may be associated with various autoimmune manifestations [4,5,6]. It may also be associated with autoimmune thyroid disease [7,8]. Autoimmune Hashimoto’s thyroiditis has been observed in patients with SLE [8]. In the context of autoimmune Hashimoto’s thyroiditis, hypothyroidism may be detected [9]. Hyperthyroidism has also been observed in patients with SLE [9,10]. However, the exact association between thyroid autoimmunity and SLE needs investigation. The aim was to investigate the relationship between thyroid disease and more specifically, autoimmune thyroid disease with SLE.

Thyroid disease has been associated with systemic autoimmune diseases [11] such as rheumatoid arthritis (RA) [12], Sjogren’s syndrome and systemic sclerosis. In particular, RA, a frequent progressive, systemic autoimmune disease characterized by chronic inflammation affecting multiple joints with associated systemic manifestations and a worldwide prevalence of 0.5–1%, has been associated with thyroid autoimmunity [13]. Various studies have estimated the presence of thyroid hormone dysfunction and autoimmune thyroid disease in RA to be between 6 and 33% [14]. In a hospital-based observational, descriptive study performed in RA patients in India, thyroid dysfunction was observed in 20% of patients [14]. The most common thyroid disorder was overt hypothyroidism followed by subclinical hypothyroidism and subclinical hyperthyroidism. Thyroid peroxidase antibodies were present in most of the patients with RA and overt hypothyroidism [14]. Sjogren’s syndrome has also been strongly associated with thyroid autoimmunity [15,16]. Autoimmune thyroid disease has been found in systemic sclerosis [17]. Various potential factors may be involved in the pathogenesis of autoimmune thyroid disease such as genetic susceptibility, environmental factors, some drugs, iodine and selenium, infections, molecular mimicry and various immune system defects [18]. However, the triggering factor that is responsible for the breakdown of immune tolerance is very difficult to discern. Despite this, the introduction into clinical practice of immune checkpoint inhibitors [19] and the emergence as major adverse effect of these agents, either de novo thyroid autoimmunity or aggravation of already existing Hashimoto’s thyroiditis has shed new light onto the pathogenesis of autoimmune thyroid disease [20]. In SLE pathogenesis, both B and T lymphocytes are involved [21,22], and the disease is a non-organ-specific disorder, as it affects all organ systems [1]. In this context, thyroid involvement may be observed [23]. The aim was to evaluate the relationship between SLE and thyroid autoimmunity in a cohort of lupus patients.

## 2. Materials and Methods

A cohort of 45 patients with SLE were consecutively and prospectively studied as they came for evaluation (Table 1). All of the patients fulfilled the 2019 EULAR/ACR criteria for the diagnosis of SLE. Serum was collected for thyroid function determination and for the measurement of thyroid peroxidase (TPOab) and thyroglobulin (Tgab) antibodies. A group of 45 control patients was evaluated as well. None of the female patients or the controls was pregnant when entering the study. The study was approved by the ethical committee of Asclepeion Hospital (approval number 2377, 23 November 2021. All of the patients and the controls gave their informed consent before entering the study.

The TSH levels were measured in sera via the ARCHITECT TSH immunoassay (Abbott Park IL, USA). The assay is a chemiluminescent microparticle immunoassay with an analytical sensitivity of <0.0025 μIU/mL, a precision of <10% and an interassay coefficient of variation of <20%. The ARCHITECT TSH assay is a two-step immunoassay that utilizes chemiluminescent microparticle immunoassay technology with flexible assay protocols, which are referred to as Chemiflex. In the first-step sample, anti-β TSH antibody-coated paramagnetic microparticles and TSH assay diluent were combined. The TSH present in the sample was bound to the anti-TSH antibody-coated microparticles. After washing, anti-α TSH acridinium-labeled conjugate was added in the second step. Pre-trigger and trigger solutions were then added to the reaction mixture; the resulting chemiluminescent reaction was measured as relative light units. A direct relationship exists between the amount of TSH in the sample and the relative light units detected by the ARCHITECT i optical system.

The free T_3_ (FT_3_) levels were measured using the ARCHITECT FT_3_ assay. The ARCHITECT FT_3_ is a chemiluminescent microparticle immunoassay (Abbott Park IL, USA). The immunoassay displays an analytical sensitivity and an analytical specificity of <1.0 pg/mL and <0.001%, respectively. The ARCHITECT free T_3_ assay is a two-step immunoassay. It serves in the determination of free T_3_ in human serum and plasma using chemiluminescent microparticle immunoassay (CMIA) technology. It utilizes flexible assay protocols. In the first step, the sample and anti-T_3_-coated paramagnetic microparticles were combined. Free T_3_ (unbound) in the sample bonded to the anti-T_3_-coated microparticles. After washing, T_3_ acridinium-labeled conjugate was added in the second step. Pre-trigger and trigger solutions were then added to the reaction mixture. The chemiluminescent reaction that took place was measured as relative light units. The assay is based on the inverse relationship that exists between the amount of FT_3_ in the sample and the relative light units detected by the ARCHITECT i optical system.

The free T_4_ (FT_4_) levels were measured with the ARCHITECT FT_4_ assay, which is a chemiluminescent microparticle immunoassay (Abbott Park IL, USA) with an analytical sensitivity of <0.4 ng/dL and a precision of <10%. The ARCHITECT free T_4_ assay is a two-step immunoassay. It is applied for the determination of the presence of free thyroxine (free T_4_) in human serum and plasma. It uses chemiluminescent microparticle immunoassay (CMIA) technology with flexible assay protocols. In the first step, the sample and anti-T_4_ coated paramagnetic microparticles were combined. FT_4_ (unbound) present in the sample bonded to the anti-T_4_ coated microparticles. After washing, T_4_ acridinium-labeled conjugate was added in the second step. Pre-trigger and trigger Solutions were then added to the reaction mixture; the resulting chemiluminescent reaction was measured as relative light units. The assay is based on the inverse relationship that exists between the amount of free T_4_ in the sample and the relative light units detected by the ARCHITECT i optical system.

The determination of TPOab was performed with the ARCHITECT anti-TPO assay (Abbott Park IL, USA), with a precision of <10% for samples >5.61 IU/mL and a within run CV of 3.9% at a concentration of 1.56 IU/mL. The ARCHITECT anti-TPO assay is a two-step immunoassay for the quantitative determination of anti-TPO in human serum and plasma using CMIA technology with flexible assay protocols, referred to as Chemiflex^®^. In the first step, the sample, assay diluent and TPO-coated paramagnetic microparticles were combined and incubated. After washing, anti-human IgG acridinium-labeled conjugate was added in the second step. Following another incubation and washing, pre-trigger and trigger solutions were added to the reaction mixture. The resulting chemiluminescent reaction was measured as relative light units. A direct relationship is observed between the amount of TPOab in the sample and the relative light units detected by the ARCHITECT i* system optics.

TgAb was assayed using the ARCHITECT anti-Tg assay (Abbott Park IL, USA). The ARCHITECT anti-Tg assay is a two-step immunoassay for the quantitative determination of the IgG class of thyroglobulin autoantibodies (anti-Tg) in human serum and plasma using CMIA technology with flexible assay protocols, referred to as Chemiflex^®^. In the first step, the sample, assay diluent and Tg-coated paramagnetic microparticles were combined and incubated. Anti-Tg present in the sample bonded to the Tg-coated microparticles. After washing, anti-human IgG acridinium-labeled conjugate was added in the second step. Following another incubation and washing, pre-trigger and trigger solutions were added to the reaction mixture. The resulting chemiluminescent reaction was measured as relative light units. A direct relationship exists between the amount of anti-Tg in the sample and the relative light units detected by the ARCHITECT i* system optics.

All of the patients underwent a complete hematological, biochemical and immunological laboratory evaluation. All of them had a chest x-ray, and in all of them, the thyroid was examined by palpation. Clinical criteria were used for the evaluation of the thyroid status as well as the thyroid hormone profile. Clinical criteria were applied for the diagnosis of hyperthyroidism (weight loss, heat intolerance, tachycardia), hypothyroidism (weight gain, cold intolerance, bradycardia) and thyroid hormone measurement. Normal FT_3_ and FT_4_ values but elevated TSH levels were consistent with subclinical hypothyroidism.

Statistical evaluation of the results was performed using the SPSS statistical package (Statistical Package for the Social Sciences, IBM SPSS v27, New York, NY, USA).

## 3. Results

### 3.1. Clinical Thyroid Disease in the SLE and Control Groups

**Figure 1:**
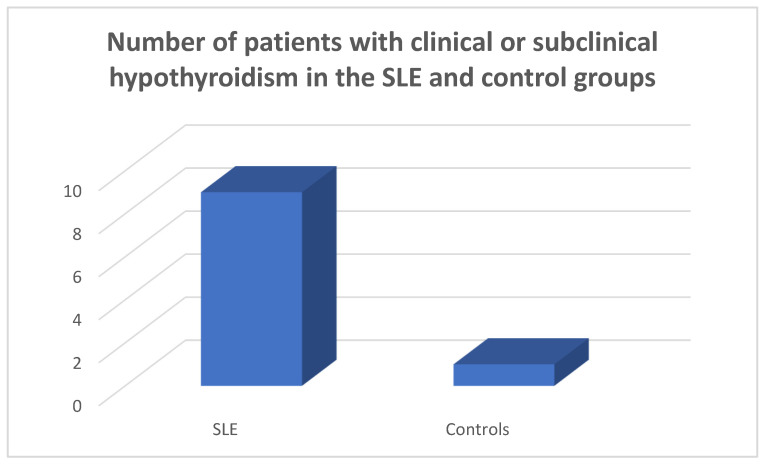
Number of patients suffering from clinical and subclinical hypothyroidism in the SLE and the control groups.

**Figure 2:**
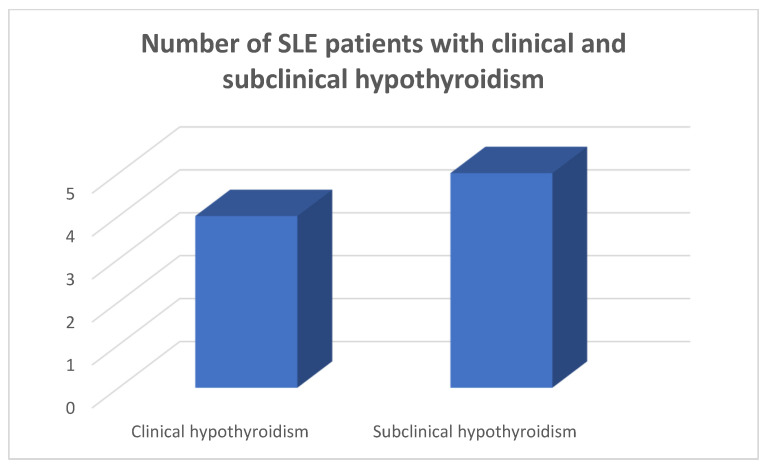
Number of SLE patients with clinical and subclinical hypothyroidism.

**Figure 3:**
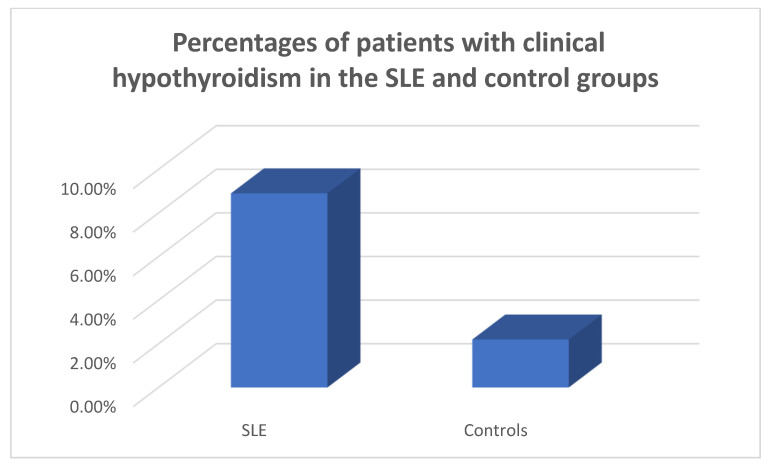
Percentages of patients with clinical hypothyroidism in the SLE and control groups.

In the patients and the controls, the mean FT_3_, FT_4_ and TSH values were within the normal range. Four patients (8.9%) were found to suffer from primary clinical hypothyroidism, five (11.11%) from subclinical hypothyroidism (Figure 1, Figure 2 and Figure 3), as opposed to one (2.22%) with hypothyroidism in the control group (chi-square test *p* = 0.0073, Fisher’s exact test = 0.015). Within the SLE group, one patient had hyperthyroidism (2.22%) and one of the controls (2.22%) had hyperthyroidism (chi square test *p* > 0.05, Fisher’s exact test = 1, *p* > 0.05). Five patients (11.11%) had a thyroid hormone profile compatible with the presence of euthyroid sick syndrome.

### 3.2. Antithytoid Antibodies in the SLE and Control Groups

**Figure 4:**
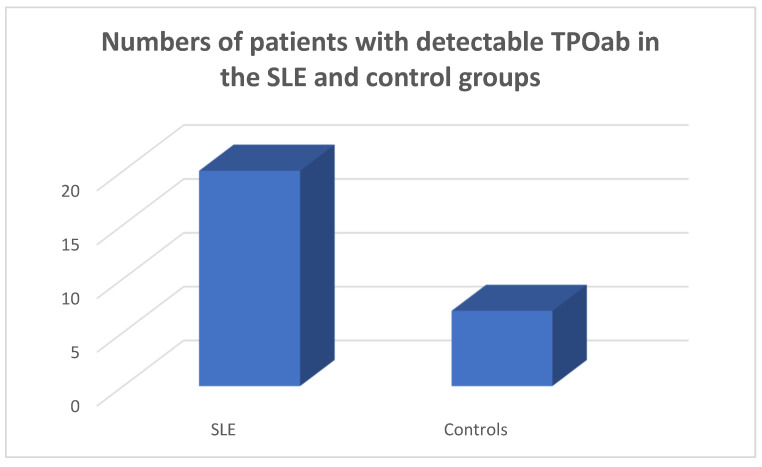
Numbers of SLE patients and controls with detectable TPOab, chi square test *p* = 0.0028, Fisher’s exact test = 0.0052.

TPOab were detected in 20/45 of the SLE population and in 7/45 of the controls (chi-square test *p* = 0.0028, Fisher’s exact test = 0.0052) (Figure 4).

**Figure 5:**
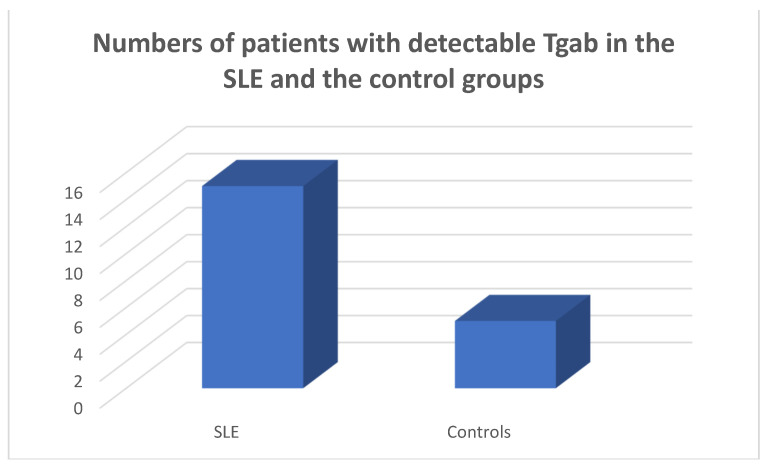
Numbers of SLE patients and controls with detectable Tgab, chi-square test *p* = 0.0028, Fisher’s exact test = 0.021.

Tgab were detected in 15/45 of the SLE population and in 5/45 of the controls, respectively (chi-square test *p* = 0.011, Fisher’s exact test = 0.021) (Figure 5).

Amongst the Tgab-positive SLE patients 3 were hypothyroid, 2 had subclinical hypothyroidism and 10 were euthyroid. Amongst the TPOab-positive SLE patients, three had hypothyroidism and two had subclinical hypothyroidism. Amongst the SLE patients who had both types of detectable antithyroid antibodies, two were hypothyroid and two had subclinical hypothyroidism.

The presence of hypothyroidism, clinical or subclinical, and the presence of TPOab and Tgab was not related to SLE disease activity as it was assessed via the SLEDAI-2K. The presence of clinical thyroid disease TPOab and Tgab was not related to organ involvement in the SLE patients.

## 4. Discussion

In a prospective 12-month study of 45 Greek SLE patients, a significantly higher prevalence of Tgab and TPOab was observed compared to a control population. Amongst the SLE patients, four had overt clinical primary hypothyroidism, five had subclinical primary hypothyroidism and one patient had hyperthyroidism. Five patients had low FT3 levels, low FT4 and normal TSH levels, a hormone profile compatible with the presence of euthyroid sick syndrome.

There are previous reports showing that patients with SLE have a higher prevalence of thyroid disease than a control population. In a very early study published in 1961, Hijmans et al. [24] detected thyroid antibodies in SLE patients at about three times the frequency of that observed in age- and sex-matched controls. In an earlier study including 332 SLE patients, Miller et al. [25] found a prevalence of hypothyroidism in 6.6% and increased antimicrosomal antibodies in 18%. In a study of 150 patients with SLE, Kausman and Isenberg [23] found 21% were positive for thyroid autoantibodies. They observed fluctuations in thyroid autoantibody levels over time. They also observed clinical thyroid disease in some of the patients with positive thyroid autoantibodies. In a study performed in Singapore involving 129 SLE patients, Boey et al. [26] found increased thyroid antibodies in 32.2% and clinical thyroid disease in some of the patients. In a study performed in Korea involving 63 lupus patients, Park et al. [27] found positive thyroid antibodies in 27%. In an early study, increased TPO antibodies were observed in the sera of SLE patients [28]. In a seminal study performed in India [29] involving a cohort of 100 SLE patients and 100 controls, 30% of the lupus patients displayed positive thyroid antibodies as opposed to 10% of the controls. Increased disease activity, as demonstrated by the SLEDAI index, was associated with the presence of euthyroid sick syndrome. Mulhern et al. [30], in a retrospective study, found no correlation between SLE and Hashimoto’s thyroiditis. Goh and Wang [31] in a study of 319 SLE Asian patients found a higher incidence of Graves’ disease than in the general population. Byron and Mowat [32] in a study in Oxford found that amongst 64 patients, 61 females and 3 males had SLE, and 10 females had thyroid disease. Amongst these, 10 SLE patients had hyperthyroidism (11.5%) and 3 had hypothyroidism (4.9%), whereas in the British population as a whole, the incidences of hyperthyroidism and hypothyroidism were 1.9% and 1%, respectively. In a more recent study performed in Brazil, Posselt et al. [7] studied a cohort of 301 SLE patients and 140 controls for the presence of antithyroid antibodies and Hashimoto thyroiditis. They observed a 12.6% prevalence of Hashimoto’s thyroiditis in lupus patients, as compared to 5.6% in the control population. They also observed that lupus patients with Hashimoto’s thyroiditis were characterized by the presence of less facial rash. Additionally, they found that lupus patients with Hashimoto’s were characterized by the detection of more anti-Sm antibodies. In this cohort, anti-Sm antibodies were more common in the group of lupus patients with both thyroid antibodies detectable. They also observed an absence of an association between Hashimoto’s thyroiditis and lupus disease activity or cumulative lupus damage. They concluded that there is a two-fold augmented risk of Hashimoto’s thyroiditis in lupus. In a study performed in China, Liu et al. [33] collected clinical, laboratory and immunologic data related to 63 patients with lupus and Hashimoto’s thyroiditis. They observed a negative correlation between FT_3_ levels and lupus disease activity, and a negative correlation between Tgab and the complement component C4. In a study performed in Hungary, Szanto et al. [34] studied a group of 56 SLE patients who also had Sjogren’s syndrome, and compared them with 50 patients with SLE and 50 patients with Sjogren’s syndrome. The researchers found an increased prevalence of thyroiditis in the group of patients with both disorders. In a study performed in China, Liu et al. [33] investigated the relationship between SLE and hypothyroidism using complementary genetic approaches, such as genetic correlation and colocalization analysis. The linkage disequilibrium score revealed a shared genetic structure between SLE and primary hypothyroidism. In a Mendelian randomization study using data from genome-wide association studies of SLE and thyroid disease in people with European ancestry, the causal link between SLE and thyroid disease was assessed [9]. The Mendelian randomization analysis showed a relationship between SLE and an increased incidence of hypothyroidism and hyperthyroidism. The sensitivity analysis of the study did not reveal any pleiotropy or heterogeneity. By contrast, Qin et al. [35], in a study using Mendelian randomization analysis, found an association between SLE and hypothyroidism, but did not find an association between SLE and hyperthyroidism. The authors performed a two-step analysis using univariable and multivariable Mendelian randomization analysis in three genome-wide association studies’ datasets. The authors concluded, based on this analysis, that SLE is associated with hypothyroidism but not with hyperthyroidism.

Thyroid disease has been associated with systemic autoimmunity [36]. In particular, thyroid disease has been associated with RA, progressive systemic sclerosis and other connective tissue disorders [36,37]. In a study performed in Russia, Odin et al. [38] studied 53 patients, 92% female, with both RA and autoimmune thyroiditis, and described some subsets of RA, such as RA occurring during the active reproductive period and late-onset RA affected by autoimmune thyroiditis. Various studies have confirmed an augmented prevalence of autoimmune thyroid disease in RA patients. This finding is in accordance with the idea that autoimmune conditions may emerge in the same patient and in families, a finding which may be related to a defect in immune tolerance [39]. Both the prevalence of autoimmune thyroid disease in RA and that of RA in autoimmune thyroiditis is increased by 1–6-fold and 1–3-fold, respectively [37]. Various early cross-sectional and observational studies have observed a prevalence of thyroiditis in RA patients of 12% [40]. RA is a common systemic autoimmune disease associated with autoimmune thyroid disease [12]. An increased prevalence of antithyroid antibodies, both TPOab and Tgab, has been observed in RA patients ranging from 5–37% and 5–31%, respectively, while both types of antithyroid antibodies have a recorded prevalence of 4% to 32% [41,42,43]. In a study performed in China, a higher prevalence of antithyroid antibodies was observed in seropositive as opposed to seronegative RA [44]. It is interesting to note that a study from China found a significantly higher prevalence of positive tests for aTPO and aTg in patients with RF as compared to those who were seronegative for RF [41]. The association between systemic sclerosis and autoimmune thyroid diseases has been evaluated by Fallahi et al. [17]. Systemic sclerosis is a connective tissue disorder characterized by microvascular involvement, immune activation and fibrosis [45]. In systemic sclerosis, autoimmune thyroiditis and hypothyroidism have been noted with an increased incidence and prevalence, especially in female patients [17]. Graves’ disease has also been noted in patients with systemic sclerosis [46].

The pathogenesis of Hashimoto’s thyroiditis is related to the production of antithyroid antibodies, namely TPOab and Tgab, with attendant lymphocytic infiltration by B and T lymphocytes. It is theorized that amongst the first events in the pathogenesis of Hashimoto’s thyroiditis is a functional alteration of B lymphocytes, leading to the production of autoantibodies [47,48]. Subsequently, T cell dysfunction is associated with the breakdown of immune homeostasis against thyroid tissue. Serum TPOab are considered the most important feature of Hashimoto’s thyroiditis, and are detectable in more than 95% of the cases [49]. By contrast, Tgab are observed in 60–80% of the cases, and are less reliable for diagnosis [50]. It is thought that Tgab represent an index of an initial immune response, whereas TPOab represent an immune escalation [51]. However, both types of antithyroid antibodies are not entirely specific for Hashimoto’s thyroiditis, and are present in other autoimmune conditions as well [52,53,54].

Hashimoto’s thyroiditis may be the first autoimmune process that emerges in a susceptible individual [55]. However, in the cohort of SLE patients described in this study, the development of thyroid autoimmunity and clinical thyroid disease was observed during the course of lupus. Both SLE and autoimmune Hashimoto’s thyroiditis share several characteristics. Both diseases occur in a susceptible genetic background when environmental factors are present, such as infections or stressful situations [18,56]. Both diseases affect preferentially female patients, as female patients have a stronger immune response with the cost of increased susceptibility to autoimmunity [57,58]. In both diseases, both B and T lymphocytes are involved in the pathogenetic process [18,56]. It appears that thyroid autoimmunity occurs in SLE in the process of multiple organ involvement, as lupus is a systemic autoimmune disease. Ferrari et al. [59] reviewed the subject of thyroid disease in SLE patients and concluded that female patients with lupus are susceptible to the development of clinical autoimmune thyroid disease, and should be periodically screened and followed-up for the development of clinical thyroid disease. Risk factors for the development of clinical thyroid disease in these patients is the detection of thyroid antibodies and thyroid hypoechogenicity on ultrasound, as well as small thyroid size. In addition, appropriate treatment for the clinical thyroid disease should be administered to these patients. In our study, we observed thyroid autoimmunity, both clinical and subclinical, in a cohort of lupus patients. The patients developed thyroid autoimmunity in the course of their systemic autoimmune disease, i.e., in the course of lupus. Patients with clinical thyroid disease were treated with the administration of either thyroxine or antithyroid medications accordingly, and they were followed up for their condition in the long term. A clinical sign of major significance in the course of lupus that should be a sign for thyroid screening is fatigue, as it affects both hypothyroid and hyperthyroid patients.

In conclusion, in a cohort of SLE patients, antithyroid antibodies were detected and thyroid disease was diagnosed. It appears that SLE patients should be screened and followed up with for the presence of thyroid autoimmunity and thyroid disease.

## 5. Conclusions

It appears that SLE patients may develop antithyroid antibodies and may present with thyroid disease. Thus, lupus patients should be screened and followed up with for the presence of thyroid autoimmunity and thyroid disease.

## Figures and Tables

**Table 1:** Characteristics of SLE patients and controls: age in years (mean ± SEM) and disease duration years (mean ± SEM). Leucopenia was defined as white blood cells < 4000/μL.

	SLE Patients
Age	47.97 ± 2.17
Sex	41 F/4 M
SLE disease duration	5.71 ± 0.49
SLEDAI-2K	9.24 ± 0.65
Leucopenia	35%
Cutaneous involvement	32%
Renal involvement	28.9%

## Data Availability

The data are available in the database of Asclepeion Hospital, Voula, Athens, Greece.
